# Prevalence of dry eye syndrome in residents of surgical specialties

**DOI:** 10.1186/s12886-016-0292-3

**Published:** 2016-07-16

**Authors:** José Alberto Castellanos-González, Verónica Torres-Martínez, Adriana Martínez-Ruiz, Clotilde Fuentes-Orozco, Jorge Rendón-Félix, Leire Irusteta-Jiménez, Aída Rebeca Márquez-Valdez, José Antonio Cortés-Lares, Alejandro González-Ojeda

**Affiliations:** Ophthalmology Department, Specialties Hospital - Western National Medical Center, Mexican Institute of Social Security, Guadalajara, Jalisco Mexico; Research Unit in Clinical Epidemiology, Specialties Hospital - Western National Medical Center, Mexican Institute of Social Security, Av. Belisario Dominguez 1000, Col. Independencia, Guadalajara, Jalisco 44240 Mexico

**Keywords:** Dry eye syndrome, Medical residency, Surgical specialties

## Abstract

**Background:**

The aim of this study was to determine the prevalence and severity of dry eye syndrome in a group of Mexican residents of different surgical specialties.

**Methods:**

A cross-sectional descriptive study where the residents were studied using the Ocular Surface Disease Index, together with diagnostic tests for dry eye syndrome, such as tear breakup time, Oxford Schema, Schirmer’s test I, and meibomian gland dysfunction testing. Statistical analyses were performed by Pearson’s chi-squared test for categorical variables and student’s *t*-test for quantitative variables. Any *P* value < 0.05 was considered statistically significant.

**Results:**

One hundred and twenty-three residents were included (246 eyes); 90 (73 %) were male and 33 (27 %) were female. The mean age was 27.8 ± 2.1 years. A higher number of residents with dry eye syndrome was found in the cardiothoracic surgery (75 %) and otorhinolaryngology (71 %) specialties; 70 % of them reported ocular symptoms, with teardrop quality involvement in >50 % of them.

**Conclusions:**

We found a prevalence of 56 % for mild-to-moderate/severe stages of the condition. Their presence in the operating room predisposes surgical residents to dry eye syndrome because of environmental conditions.

## Background

Dry eye syndrome (DES) is recognized as a growing public disease and is one of the most frequent causes of ophthalmological consultation [1, 2]. According to the International Dry Eye Workshop (DEWS) 2007, DES is defined as a multifactorial disease of the tears and ocular surface that results in symptoms of discomfort, visual disturbance, and tear-film instability, with potential damage to the ocular surface. It is accompanied by increased osmolarity of the tear film and inflammation of the ocular surface [1, 3–6].

DES affects millions of people worldwide (10–20 % of the population), with a negative effect on their quality of life [7].

DES results in dryness of the conjunctiva and cornea because of decreased function of the tear and rapid evaporation. These patients typically have damage of the ocular surface epithelium and a deficiency in lacrimal secretion, with chronic ocular irritation symptoms [8]. They complain mainly of a persistent dry eye sensation, grittiness, ocular irritation, pain, tingling, foreign body sensation, and visual disturbances [1, 4, 9, 10], which affect their ability to work, read, use a computer, and drive at night [4, 11]. Several studies have demonstrated that in the presence of DES, either mild or severe, the quality of life is diminished [4, 5, 12], to a level that is comparable with the quality of life of patients undergoing dialysis, severe angina, and hip fracture [5, 13].

There is an increase in the prevalence of DES in relation to the number of working hours spent using a digital device, which is significant among individuals who are exposed for 2–4 h per day [11, 14].

The Women’s Health Study (WHS) and the Physicians’ Health Study (PHS), which were performed in the North American population, estimated that about 4.61 million Americans aged 50 years or older had DES, among whom 3.23 million were female and 1.68 million were male. According to the WHS, there is a higher prevalence of severe symptoms and/or DES in Hispanic and Asian women compared with Caucasians [7]; moreover, Ellwein et al. found an increase of 57.4 % in the incidence rate of DES per 100 beneficiaries of medical services, from 1.22 in 1991 to 1.92 in 1998. The importance of DES in public health is given by the high prevalence of this disease in the late-adulthood age group [15].

There is currently no data about the prevalence of dry eye syndrome in healthcare personnel. Residents of surgical specialties are exposed to risk factors that condition them to the development of ocular symptoms, because most of their training occurs inside the operating room (OR), where the ventilation environment is enclosed and the accuracy of the procedures to be performed as well as the use of a microscope require greater concentration and fixation on details, which decreases the blink rate, causing damage to the ocular surface. As a consequence, the presence of trigger factors affects the performance of the physician-in-training because of how it impacts visual function and general welfare. Therefore, the purpose of this study was to determine the prevalence of DES in residents of the surgical branches of the Specialty Hospital, Western National Medical Center.

## Methods

A cross-sectional descriptive study in medical residents of the different surgical branches of the Specialties Hospital, Western National Medical Center, Mexican Institute of Social Security, was performed from February through December 2014. The surgical specialties assessed were angiology, cardiothoracic surgery, general surgery, coloproctology, neurosurgery, ophthalmology, oncological surgery, otorhinolaryngology, traumatology and orthopedics, and urology. Surgical residents of any year participated in this study. The environmental conditions of the OR to which residents were exposed, had a 64–75 °F room temperature, and 55 % humidity.

The Ocular Surface Disease Index (OSDI) questionnaire about associated risk factors was administered, as well as diagnostic tests such as tear breakup time (TBUT), Oxford Schema, Schirmer’s test I, and meibomian gland dysfunction testing. All residents were requested to agree to participate in the study with a written informed consent.

### Ocular surface disease index (OSDI)

The OSDI questionnaire was used to measure DES based on three symptomatic subscales, referring to the week prior to the implementation of the questionnaire. The possible answers were: always, almost always, half the time, sometimes, and never. The scales evaluated were ocular discomfort, functionality, and environmental factors. A sum score on a 0–100 scale was obtained and classified as: normal (0–12), mild (13–22), moderate (23–32), or severe (33–100).

### Questionnaire about risk factors

This questionnaire included data about sex, age, surgical residency and current year, ocular and systemic diseases, allergies, use of contact lenses, medication, hormonal contraceptives, smoking, makeup use, and average hours per day spent in the OR. In addition, the daily time spent in the OR during a working day of 8 h and 24 h on-call duty was evaluated; the use of a microscope for surgical procedures and its average daily use (0–2 h, 2–4 h, and >4 h) were also assessed.

### Slit lamp biomicroscopy

#### Tear breakup time (TBUT)

TBUT was defined as the interval between the last complete blink and the appearance of the first dry spot. A sterile fluorescein strip (1 mg Fluorescein Sodium Ophthalmic strip, USP BioGlo™ Sterile Strips, HUB Pharmaceutics LLC, Rancho Cucamonga, CA) was placed in the lower tarsus, and the resident was asked to blink several times loosely 10 s after the instillation of fluorescein. The slit lamp was set to a 10× magnification using a cobalt blue light filter. A TBUT >10 s was defined as normal, and a TBUT ≤10 s was defined as dry eye.

#### Assessment of ocular surface damage by staining—Oxford schema

Using the Oxford Schema, which was validated by the DEWS 2007, staining was represented by a dotted line in a series of panels (A–E). Another strip of fluorescein was used for this test; it was instilled with a drop of saline solution, the excess was removed, and it was placed in the lower tarsal conjunctiva by pulling the lower eyelid. The same procedure was used in both eyes. Staining was evaluated under a cobalt blue light filter; to evaluate the cornea, the upper lid was lifted to include the total area of the cornea.

#### Schirmer’s test I

The test consisted of a drop instillation of 0.5 % tetracaine hydrochloride ophthalmic solution with a 2-min waiting time before the test. After wiping out the excess tear, we proceeded to the placement of a Schirmer’s strip at the junction of the lateral 1/3 and medial 2/3 of the lower lid margin. Slow eyelid movements were allowed during the procedure. Moisture was considered normal if the strip was moistened over 10–30 mm, and hyposecretion was considered in cases of strip moistening less than 10 mm.

#### Meibomian gland dysfunction testing

Gland dysfunction was registered using a test validated by The International Workshop on Meibomian Gland Dysfunction (2011). Finger pressure was applied to the nasal third of each eye’s lower lid. Meibomian gland secretion, or “meibum”, quality was assessed in each of the 8 glands of the central third of the lower lid, on a 0–3 scale for each gland: 0 = clear meibum; 1 = cloudy meibum; 2 = cloudy with debris (granular); 3 = thick, like toothpaste. The expressibility of the meibum was assessed from 5 glands: 0 = all glands were expressible; 1 = 3–4 glands were expressible; 2 = 1–2 glands were expressible; 3 = no glands were expressible. Gland dysfunction was classified according to the score obtained:

- *Stage 1* = minimally altered secretions: grade >2 – <4; expressiveness: 1.
- *Stage 2* = moderately altered secretions: grade >4 – <8; expressiveness: 1.
- *Stage 3* = moderately altered secretions: grade >8 – <13; expressiveness: 2.
- *Stage 4* = severely altered secretions: grade >13; expressiveness: 3.

### Statistical analysis and data processing

The data were captured in a database and were analyzed using the SPSS software, version 22.0 (IBM, Armonk, NY, 2013). The prevalence and range of the independent variable was estimated, with a confidence interval of 95 %. Statistical analyses were performed by Pearson’s chi-squared test for categorical variables and Student’s *t*-test for quantitative variables. Any *P* value < 0.05 was considered statistically significant.

### Sample size

Sample size for cross-sectional studies was assessed considering an alpha of 0.05 and a beta of 0.20 using Stalcalc in Epi Info™, version 6.0 (Atlanta: Centers for Disease Control and Prevention; 1994), in which it was estimated to find DES in 30 % of the residents of the surgical area, where 125 residents were required. The confidence level was 95 %, and the power was 80 %.

## Results

One hundred and twenty-five residents (250 eyes) who met the established criteria were assessed. Two residents were excluded for presenting ocular disease during the previous week, leaving a total of 123 residents (246 eyes), of whom 90 (73 %) were male and 33 (27 %) were female. The mean age was 27.8 ± 2.1 years. Two physicians outside of the study conducted the Schirmer’s I test and the TBUT. Inter-observer variation coefficients for the measurements were evaluated in 123 residents for both tests, resulting in 1.3 % and 1.2 %, respectively.

The prevalence of DES was higher in the female population (22, 67 %) than it was in the male population (47, 52 %). Based on the OSDI, 56 % of the assessed residents presented DES and 44 % were at a normal stage. Among them, 21 % had mild, 18 % had moderate, and 17 % had severe ocular symptoms. Of the 11 surgical specialties evaluated, cardiothoracic surgery (75 %) and otorhinolaryngology (71 %) presented a higher number of residents with a positive OSDI test (either mild, moderate, or severe).

Among the four diagnostic tests applied, the TBUT test showed that the quality of the tear was affected in more than 50 % of the evaluated eyes: the TBUT was <10 s in 57 % of the residents. A significant difference was found in the first and seventh year of residency (*P* = 0.008; *P* = 0.01, respectively). According to the Oxford Schema, 76 % of the residents were classified as grade 0, whereas the remaining 24 % were grade I (slight staining of the ocular surface). Residents in their fourth year showed a significant difference (*P* < 0.001). No grade II, III, IV, or V conditions were observed. The results of TBUT and Oxford Schema testing in relation to chronic eye disease, use of contact lenses, refractive surgery, risk factors, use of makeup, and use of a microscope are listed in Table 1.

**Table 1:** Tear breakup time and Oxford Schema in relation to risk factors

Variable	Tear breakup time <10 s	Tear breakup time >10 s	*P* value	Oxford Schema 0	Oxford Schema 1	*P* value
1^st^ year of residency	33 (79 %)	9 (21 %)	0.008	28 (67 %)	14 (33 %)	0.06
2^nd^ year of residency	34 (63 %)	20 (37 %)	0.88	45 (83 %)	9 (17 %)	0.54
3^rd^ year of residency	32 (47 %)	36 (53 %)	0.14	58 (85 %)	10 (15 %)	0.29
4^th^ year of residency	23 (68 %)	11 (32 %)	0.23	22 (65 %)	12 (35 %)	<0.001
5^th^ year of residency	7 (32 %)	15 (68 %)	0.02	19 (86 %)	3 (14 %)	0.33
6^th^ year of residency	10 (56 %)	8 (44 %)	0.91	16 (89 %)	2 (11 %)	0.27
7^th^ year of residency	1 (12 %)	7 (88 %)	0.01	8 (100 %)	0 (0 %)	0.16
Chronic eye disease	43 (58 %)	31 (42 %)	0.80	62 (84 %)	12 (16 %)	0.29
Contact lenses	13 (81 %)	3 (19 %)	0.04	9 (56 %)	7 (44 %)	0.02
Refractive surgery	19 (68 %)	9 (32 %)	0.21	24 (86 %)	4 (14 %)	0.39
Allergies	41 (66 %)	21 (34 %)	0.09	52 (84 %)	10 (16 %)	0.34
Smoking	21 (50 %)	21 (50 %)	0.32	32 (76 %)	10 (24 %)	0.53
Hormonal contraceptives	9 (56 %)	7 (44 %)	0.10	14 (87 %)	2 (13 %)	0.51
Makeup use	41 (71 %)	17 (29 %)	0.66	51 (88 %)	7 (12 %)	0.04
Makeup use, 0–12 h	23 (68 %)	11 (32 %)	0.54	34 (100 %)	0 (0 %)	0.001
Makeup use, 12–24 h	18 (75 %)	6 (25 %)	0.54	17 (71 %)	7 (29 %)	0.001
Microscope use	73 (68 %)	35 (32 %)	0.10	84 (78 %)	24 (22 %)	0.003

In contrast, in the Schirmer’s test I, 100 % of eyes exhibited normal parameters. Meibomian gland dysfunction testing reported that 77 % of the residents were in stage 1 (normal), whereas the remaining 23 % of the residents were in stage 2 (mild meibomian gland dysfunction). According to year of residency, a significant difference was found in first and fourth year residents (*P* = 0.003; *P* = 0.001, respectively). No eyes were classified as being in stages 3 or 4. Regarding risk factors, there was a significant difference in dysfunction of the meibomian glands in smokers (*P* = 0.009). The results of the Schirmer’s and meibomian gland dysfunction tests in relation to chronic eye disease, use of contact lenses, refractive surgery, risk factors, use of makeup, and use of a microscope are listed in Table 2.

**Table 2:** Results of Schirmer’s I and meibomian gland dysfunction tests in relation to risk factors

Variable	Schirmer’s test +	Schirmer’s test –	Meibomian gland dysfunction stage 1	Meibomian gland dysfunction stage 2	*P* value
1^st^ year of residency	0 (0 %)	42 (100 %)	27 (64 %)	15 (36 %)	0.003
2^nd^ year of residency	0 (0 %)	54 (100 %)	42 (77 %)	12 (23 %)	0.83
3^rd^ year of residency	0 (0 %)	68 (100 %)	53 (78 %)	15 (22 %)	0.90
4^th^ year of residency	0 (0 %)	34 (100 %)	23 (68 %)	11 (32 %)	0.001
5^th^ year of residency	0 (0 %)	22 (100 %)	20 (91 %)	2 (9 %)	0.10
6^th^ year of residency	0 (0 %)	18 (100 %)	17 (94 %)	1 (6 %)	0.47
7^th^ year of residency	0 (0 %)	8 (100 %)	8 (100 %)	0 (0 %)	0.13
Chronic eye disease	0 (0 %)	74 (100 %)	61 (82 %)	13 (18 %)	0.20
Contact lenses	0 (0 %)	16 (100 %)	15 (94 %)	1 (6 %)	0.08
Refractive surgery	0 (0 %)	28 (100 %)	22 (79 %)	6 (21 %)	0.85
Allergies	0 (0 %)	62 (100 %)	47 (76 %)	15 (24 %)	0.075
Smoking	0 (0 %)	42 (100 %)	26 (62 %)	16 (38 %)	0.009
Hormonal contraceptives	0 (0 %)	16 (100 %)	8 (50 %)	8 (50 %)	0.013
Makeup use	0 (0 %)	58 (100 %)	27 (47 %)	31 (53 %)	0.09
Makeup use, 0–12 h	0 (0 %)	34 (100 %)	21 (62 %)	13 (38 %)	0.025
Makeup use, 12–24 h	0 (0 %)	24 (100 %)	6 (25 %)	18 (75 %)	0.025
Microscope use	0 (0 %)	108 (100 %)	73 (68 %)	35 (32 %)	0.47

Fifty-four residents (44 %) mentioned the use of a microscope during surgery, whereas 56 % (69) did not require a microscope. OSDI ranking was obtained for those who used a microscope: 37 % (20) presented a normal OSDI test, and 63 % (34) exhibited mild, moderate, or severe OSDI grading.

Regarding year of residency, first-year residents had a higher percentage of DES according to OSDI (76 % residents affected), followed by sixth-year residents (67 %), second-year residents (57 %), and third-year residents (56 %). When asked about eye symptoms (sensitivity to light, grittiness, eye pain, and blurred or poor vision) during the last year of residency, as well as their presence during surgery, on-call, or microscope use, 71 % (87) of the residents reported having them.

The relationship between operating hours in an 8 h working day and OSDI score was evaluated: >50 % of residents who operated an average of 0–6 h per day were positive for DES. According to operating hours on a 24 h on-call shift, >55 % of those with a surgery time of 0–2 h and >4 h presented a positive OSDI score for DES.

## Discussion

It is well known that DES is a growing disease, affecting approximately 10–20 % of the population worldwide [1]. In the population over 50 years of age, the prevalence of DES ranges from 5 % to 30 %; however, by 2050, the estimated prevalence will reach up to 100 % [2]. In this sense, the mean age observed in the present study was 27.8 years, and 56 % of the residents presented DES in a mild, moderate, or severe form, according to the OSDI. This fact proves the great epidemiological relevance of the development of DES at a younger age than reported previously. In the study published by Sahai et al., a peak of prevalence of DES was reported within the 31–40-year-old group, which was attributed to the type of environmental exposure [2].

Several studies reported a higher prevalence of DES in females [2, 7], similar to the results of this study, with a prevalence of 67 % (22). This difference can be explained based on the relationship between the menstrual cycle phase and the presence of ocular symptoms [6]. In physiological doses, estrogens serve as support for the function of the lacrimal gland and the preservation of a healthy ocular surface at an early age; however, in high doses or in combination with hormone supplements, they can be harmful and/or induce inflammation [16]. Forty-eight percent of the women surveyed reported using some type of hormonal contraceptive, among whom 75 % presented DES, with lacrimal gland dysfunction in >50 % of them. Another risk factor associated with the female sex is the use of makeup, because of the changes caused in the structure of the tear film by migration to the ocular surface [17], which is in keeping with our findings that 71 % of female residents showed tear-film instability and that 53 % exhibited mild meibomian gland dysfunction.

A study conducted by Sahai et al. found that patients with a refractive error had a higher prevalence of DES than did emmetropes; however, the cause of this phenomenon was not determined in that study [2]. This prevalence is similar to the one found in residents who reported either ocular conjunctivitis or ametropia, with a positive OSDI for DES. In our study, 71 % of the population that had undergone refractive surgery presented a positive OSDI for DES, among whom 68 % presented a TBUT of <10 s. This is comparable with the fact that 88 % of the residents who wore contact lenses had DES according to the OSDI test, which is consistent with previous reports in the literature [4, 18, 19]. However, regarding cigarette consumption, this study contrasts with the literature, as the presence of DES was not found in residents who smoked. Smoking is a risk factor for DES because it increases ocular symptoms by altering the tear evaporation rate [20].

In the study conducted by Smedbold et al., who assessed nurses working in different departments of geriatric hospitals with the aim of studying the relationship between the environment and signs of eye irritation, it was found that, in high-temperature environments with low humidity, the tear evaporation rate was increased, causing instability of the tear film [7, 21]. No additional studies of the presence of DES in health professionals working in hospital environments were found. However, DES has been studied well in office workers who use any digital device, with an increased prevalence in those exposed to them for 2–4 h a day [11, 14, 18]. Uchino et al. reported that a period >4 h a day working in front of a digital device increases the expression of severe symptoms of DES, with significant clinical relevance (*P* < 0.001) [11]. Seventy percent of respondents in the study presented ocular symptoms over the last year. Among them, more than half presented a positive OSDI test for DES (*P <*0.001). Moreover, 72 % presented ocular symptoms during surgery, among whom 77 % had DES (*P* = 0.002). In addition, 67 % of these individuals presented symptoms during an on-call shift, more than half of whom presented a positive OSDI test for DES (*P* <0.001).

Environmental irritants can be associated with the “sick building syndrome” (i.e., a disease caused by irritants found in the workplace, volatile organic compounds, and low-humidity conditions), in which there is an eye and mucous membrane irritation, causing an unstable tear film in office workers [22, 23]. It is well known that the climate has an influence on the clinical diagnostic dry eye tests. The present study was conducted in Guadalajara, Jalisco, Mexico with an average temperature of 65 °F (53–81 °F) that remains stable all year round, and a temperature of 64–75 °F inside the OR.

The lack of reliable diagnosis due to a bedeviled assessment of dry eye syndrome might be secondary to poor diagnostic repeatability, which is manifested as significant false-positive/negative rates, broad range of variability, wide range of sensitivity and specificity, and dependence on clinical conditions [24].

We may also consider anterior-segment optical coherence tomography as a diagnostic tool, which evaluates epithelial thickness and reveals abnormalities of the dynamic pattern of tears, even when the common lacrimal tests appear to be normal [25].

Recently, tear osmolarity has been proposed as the gold standard for DES, but there is still no consensus about its use as the best diagnostic tool. Therefore, several diagnostic modalities should be performed, such as diagnostic tests and external examination, based on the patient’s symptoms and medical history [1, 26].

We propose further comparative studies to assess timing, quality, accuracy of procedures, and evaluation to verify how DES negatively affects the performance of the residents.

## Conclusions

The presence of surgical residents in the OR renders them susceptible to the development of DES because of the environmental conditions to which they are exposed, because of which causes ocular discomfort. This condition affects the quality of life and performance of residents, because of the irritative symptoms, the modification of the visual function, and the effects on general health and welfare. Fifty-six percent of the residents surveyed presented DES.

## Abbreviations

DES, dry eye syndrome; DEWS, international dry eye workshop; OR, operating room; OSDI, ocular surface disease index; PHS, physicians’ health study; TBUT, tear break-up time; WHS, women’s health study
